# A Novel Transformer-Based Attention Network for Image Dehazing

**DOI:** 10.3390/s22093428

**Published:** 2022-04-30

**Authors:** Guanlei Gao, Jie Cao, Chun Bao, Qun Hao, Aoqi Ma, Gang Li

**Affiliations:** 1 Key Laboratory of Biomimetic Robots and Systems, School of Optics and Photonics, Beijing Institute of Technology, Beijing 100081, China; 3220200417@bit.edu.cn (G.G.); caojie@bit.edu.cn (J.C.); baochun@bit.edu.cn (C.B.); 1120193414@bit.edu.cn (A.M.); 2 Yangtze Delta Region Academy, Beijing Institute of Technology, Jiaxing 314003, China; 3 Department of Electronic and Optical Engineering Shijiazhuang, Army Engineering University of PLA, Shijiazhuang 050003, China; ligangopt@sina.com

**Keywords:** image dehazing, Transformer, convolutional neural network

## Abstract

Image dehazing is challenging due to the problem of ill-posed parameter estimation. Numerous prior-based and learning-based methods have achieved great success. However, most learning-based methods use the changes and connections between scale and depth in convolutional neural networks for feature extraction. Although the performance is greatly improved compared with the prior-based methods, the performance in extracting detailed information is inferior. In this paper, we proposed an image dehazing model built with a convolutional neural network and Transformer, called Transformer for image dehazing (TID). First, we propose a Transformer-based channel attention module (TCAM), using a spatial attention module as its supplement. These two modules form an attention module that enhances channel and spatial features. Second, we use a multiscale parallel residual network as the backbone, which can extract feature information of different scales to achieve feature fusion. We experimented on the RESIDE dataset, and then conducted extensive comparisons and ablation studies with state-of-the-art methods. Experimental results show that our proposed method effectively improves the quality of the restored image, and it is also better than the existing attention modules in performance.

## 1. Introduction

In severe weather, such as haze, fog, rain, or snow, capturing high-quality images is a challenging task due to reduced visibility. These conditions also reduce the performance of advanced vision tasks, such as image classification, object detection, and scene analysis. Therefore, it is of great significance to remove the influence of severe weather in images [1,2]. For example, image dehazing, deraining, or desnowing has received a lot of attention [3,4,5,6,7,8,9,10]. In the field of image dehazing, it can be divided into general scenes and remote sensing images. For remote sensing images, preprocessing is required by wavelet-based denoising or newer versions of compressed sensing [11,12,13]. Our work proposes an image dehazing model combining Transformers and CNNs for general scenes.

In this paper, we propose a model for image dehazing.

Image dehazing has been widely studied in recent years. Most methods implement haze removal through the atmospheric scattering model [14], as shown in Equation (1):

(1) I(x)=J(x)t(x)+A(1−t(x)),

where I(x) is the hazy image formed by the scattering medium, J(x) is the restored haze-free image, t(x) is the transmission matrix, A is the global atmospheric light, and x is the pixel position.

The purpose of haze removal is to restore the haze-free image J(x) from the hazy image *I*(*x*). If the transmission matrix t(x) and the global atmospheric light A are known, we can restore the haze-free image by Formula (1). However, in most methods, since only the transmission matrix t(x) is estimated, and the global atmospheric light A is a given fixed value given by experience, haze removal for the image is a problem of ill-posed parameter estimation. Although the atmospheric scattering model is relatively intuitive, the given global atmospheric light A has a certain error and will be accumulated, so the quality of the haze-free image may be suboptimal. Therefore, AOD-Net [15] was proposed by Li et al., which unifies the two parameters t(x) and A into one parameter, i.e., K(x), and converts the atmospheric scattering model into a model with unique parameters. However, since AOD-Net [15] is a lightweight CNN model, it does not perform well in processing detailed information.

At present, the main architecture in the field of computer vision is still based on convolutional neural networks. However, the self-attention mechanism and the Transformer architecture have achieved great success in natural language processing. Some models apply the self-attention module substitute as part of the convolutional layers in ResNet [16,17]. There are also some models that employ the self-attention mechanism or Transformer to enhance or supplement the backbone of CNNs [18,19]. Recently, the Transformer-based network architecture has been widely applied in computer vision tasks [20,21,22,23,24], and it has achieved excellent performance. It is worth noting that there is very little work using the self-attention mechanism or Transformer for image dehazing. Zhao et al. [20] proposed a hybrid local–global transformer (HyLoG-ViT) for single-image dehazing, which can capture both local and global dependencies.

Inspired by ViT [22] and SE-Net [19], we propose a Transformer-based channel attention module (TCAM), which is applied to the dehazing convolutional neural network of a single image. We use the spatial attention module behind the TCAM as its supplement. Therefore, we propose an attention module that includes two parts: TCAM and the spatial attention module. The attention module enhances features along the channel and spatial dimensions. At the same time, we use a multiscale parallel residual network as the backbone, which can extract feature information of different scales to achieve feature fusion. The network is trained on synthesized hazy images and tested on both synthetic and natural images. The experimental results show that, compared with state-of-the-art methods, our method can achieve great improvements in final restoration quality.

The contributions of this work are summarized as follows:

1. We propose to apply Transformer as a channel attention module to the image dehazing task. We perform quantitative and qualitative comparisons with state-of-the-art methods on synthetic and real-world hazy image datasets, achieving better results on both.
2. Our proposed Transformer-based channel attention module (TCAM) is a plug-and-play module that can be applied to other models or tasks, such as image classification, object detection, etc.
3. We demonstrate that our proposed attention module effectively enhances detailed information. Compared with SE-Net [19] and CBAM [18], our proposed attention module achieves 6.05% and 3.29% higher PSNR and 2.80% and 3.00% higher SSIM.

## 2. Related Work

The existing methods of haze removal can be divided into the prior-based method and the learning-based method according to different data processing methods [25].

### 2.1. Prior-Based Method

The prior-based method estimates the transmission matrix and the global atmospheric light by assuming scene and hand-crafted priors.

Fattal et al. [26] redefined the atmospheric scattering model by adding new surface shadow variables and assumed that the surface shadow and the transmission function are statistically independent. Tan et al. [27] assumed that the haze-free image has higher contrast than the hazy image, and they improved the image quality by enhancing the contrast of the image. He et al. [28] observed that at least one color channel has a very low pixel value in the local area of most haze-free outdoor images, and they proposed a dark channel prior dehazing algorithm. Tang et al. [29] studied haze-relevant priors based on the regression framework to identify the best prior combination. Berman et al. [30] observed that the pixels in the RGB space clusters of the haze-free image are usually nonlocal, and these clusters form haze lines in the hazy image. Zhu et al. [31] proposed an image dehazing framework based on artificial multiexposure image fusion, which first combines the global and local details of the gamma-corrected image, and then balances the image brightness and color saturation to obtain the corresponding haze-free image.

Since these prior-based methods use hand-crafted priors and specific scenes as preconditions, the performance of these methods in haze removal is inferior if the prior is invalid or insufficient. For example, DCP [28] is less effective in processing highlights or large white areas and sky areas.

### 2.2. Learning-Based Method

Recently, CNNs have achieved great success in the field of computer vision. Therefore, learning-based methods using CNN have been widely proposed, which solve the problem of prior-based methods that rely heavily on hand-crafted priors and restrictions on specific scenarios.

Cai et al. [32] proposed an end-to-end dehazing network named DehazeNet to predict the transmission matrix, where the global atmospheric light is a given fixed value. Ren et al. [33] proposed a model that extracts features through coarse-scale and fine-scale networks to estimate the transmission matrix, called single image dehazing via multiscale convolutional neural network (MSCNN). However, these end-to-end models only estimate the transmission matrix. If the transmission matrix or global atmospheric light is inaccurate, the quality of the restored image will be suboptimal. Li et al. [15] proposed the all-in-one dehazing network, which integrates the transmission matrix *t(x)* and the global atmospheric light A into a parameter K(x). Zhang et al. [34] proposed a model that can learn the transmission matrix and global atmospheric light separately, called densely connected pyramid dehazing network (DCPDN). It introduces a generative adversarial network to identify the restored image. Qu et al. [35] proposed a model that does not rely on the atmospheric scattering model, called enhanced Pix2pix dehazing network (EPDN). The model was composed of three modules: multiresolution generator, multiscale discriminator, and enhancer. However, the generator has certain limitations in generating real detail features. Wu et al. [36] proposed a model consisting of an autoencoder-like dehazing network and contrastive regularization, called contrastive learning for compact single-image dehazing (AECR-Net). Nevertheless, contrastive learning requires a certain proportion of negative samples, which will seriously slow down the training speed. Using the attention mechanism pays attention to detailed features without slowing down the training speed. Consequently, it is meaningful to introduce the attention module.

## 3. Proposed Network Framework

**Figure 1:**
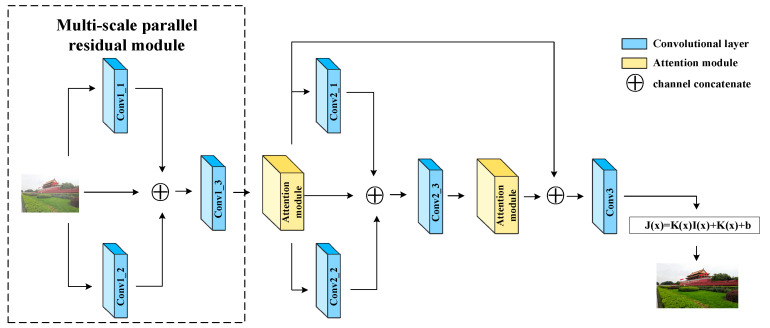
Architecture of the proposed Transformer for image dehazing (TID).

The overall architecture of our proposed Transformer for image dehazing (TID) is shown in Figure 1. The TID consists of two modules: the multiscale parallel residual module and the attention module.

### 3.1. Multiscale Parallel Residual Module

As shown in the dashed box in Figure 1, the multiscale parallel residual module [37] we used can be formulated by Equation (2).

(2) $$y=Conv_{3}(\partial (Conv_{1}(X))⊕\partial (Conv_{2}(X))⊕X),$$

where $Conv_{i}$ represents the convolutional operation of different filter sizes, ⊕ represents channel concatenate, and ∂ represents the ReLU [38] function.

Compared with the conventional residual module, the multiscale parallel residual module is used to extract multiple feature information of different scales and superimpose the original input. Here, we only extract two feature maps of different scales. Then, we input the obtained concatenated feature map of the attention module.

### 3.2. Attention Module

#### 3.2.1. Transformer-Based Channel Attention Module

**Figure 2:**
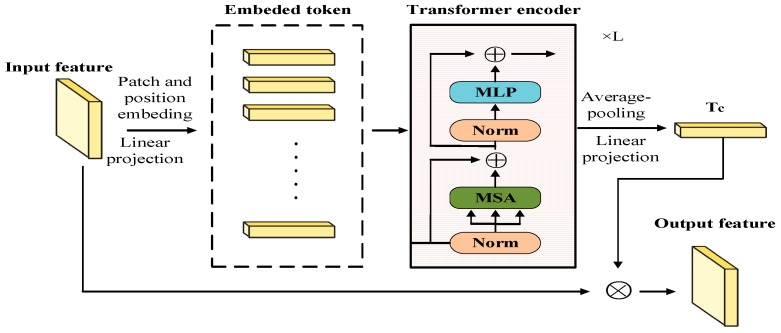
Architecture of the proposed Transformer-based channel attention module (TCAM).

The existing methods of calculating channel attention are all achieved by compressing the spatial dimension of the feature map. SE-Net [19] used the average-pooling operation to squeeze the spatial information of its feature map to obtain the channel attention map. CBAM [18] used both average-pooling and max-pooling to calculate the channel attention map. Compared with simply using average-pooling or max-pooling to squeeze the spatial dimension, we propose a module that uses the Transformer architecture to obtain channel attention, which is called the Transformer-based channel attention module (TCAM). The specific details are shown in Figure 2.

Transformer uses a one-dimensional sequence as the input token. Therefore, we need to perform patch embedding on the input feature map. First, we divide the input feature map into image patches with a resolution of P×P, and then each image patch is flattened into a one-dimensional sequence, which is called the token. If the input feature map $X_{0}\in R^{H\times W\times C}$, the token after patch embedding is $X_{t}\in R^{N\times (P^{2}•C)}$, where H, W, and C are the batch size, height, width, and channels, respectively, and $N=HW/P^{2}$ is the number of patches. Then, all tokens are linearly projected to a constant size D for the Transformer encoder to compute. In order to ensure the position information of each image patch, all tokens need to be position embedded, as shown in Equation (3).

(3) $$T_{0}{=X}_{t}+E_{p},$$

where $E_{p}\in R^{N\times D}$ is the random number generated.

$T_{0}$ is the input token of the Transformer encoder. The Transformer encoder [39] includes two-layer normalization (LN), a multihead self-attention (MSA) and a multilayer perceptron block, where LN comes before MSA and MLP. In addition, to avoid the disappearance of the gradient, the Transformer encoder uses the residual connection behind MSA and MLP [23,40]. Among them, MLP is a two-layer perceptron with expansion ratio r. The processing of the *l*-th Transformer encoder can be expressed as

(4) $$T_{l-1}^{\prime }=MSA(LN(T_{l-1}))+T_{l-1},l=1\ldots L,$$

(5) $$T_{l}=MLP(LN(T_{l-1}^{\prime }))+T_{l-1}^{\prime },l=1\ldots L,$$

where L represents the number of Transformer encoders.

Then, we perform the average-pooling (AvgPool) operation along the first dimension of the output $T_{L}$ of the Transformer encoder. The channel attention map $T_{c}\in R^{1\times C}$ is obtained by linear projection, formulated as in Equation (6).

(6) $$T_{c}=LN(AvgPool(T_{L}))E_{c},E_{c}\in R^{D\times C},$$

The output feature map of TCAM can be expressed by Equation (7).

(7) $$X_{out}=T_{c}⊗X_{0},X_{0}\in R^{H\times W\times C},$$

where ⊗ denotes pixel-level dot multiplication. In the dot multiplication, the channel attention map $T_{c}$ expands along the spatial dimension through the broadcast mechanism.

Compared with ViT [22] and DeiT [41], which use the class token as the output, our proposed TCAM performs the average-pooling operation on patch tokens to calculate the channel attention value of the feature map to achieve the purpose of enhancing the detailed information. The experimental results demonstrate that TCAM is effective.

#### 3.2.2. Spatial Attention Module

We use a spatial attention module as a supplement to TCAM. The spatial attention module from CBAM [18] performs average-pooling and max-pooling along the channel axis, and then connects the two spatial attention maps. The spatial attention map $T_{s}$ can be calculated by Equation (8).

(8) $$T_{s}=\sigma (Conv_{7\times 7}(T_{avg}⊕T_{\max})),$$

where σ denotes the sigmoid function, ⊕ represents channel concatenate, and $Conv_{7\times 7}$ represents a convolution operation with a kernel size of 7×7.

**Figure 3:**
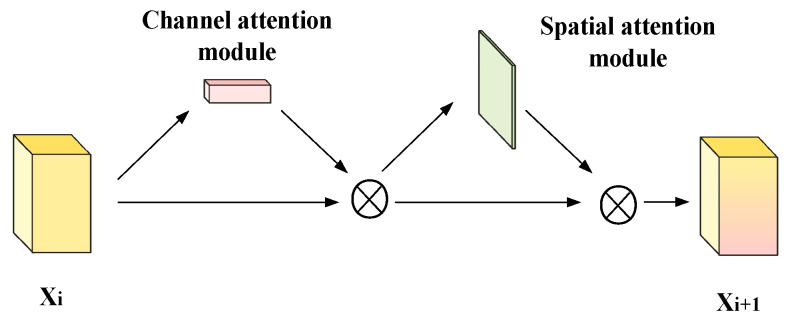
Architecture of the proposed attention module.

As shown in Figure 3, the attention module includes two parts: TCAM and the spatial attention module. This module can realize the enhancement of the detailed information of the feature map, and the overall process can be expressed by Equation (9).

(9) $$X_{i+1}=(X_{i}⊗T_{c})⊗T_{s},$$

where ⊗ denotes pixel-level dot multiplication.

The overall architecture of our proposed model is shown in Figure 1, we use two multiscale parallel residual modules and two attention modules. Finally, we concatenate the feature maps output by the two attention modules along the first dimension, and then obtain K(x) through a convolutional layer. Table 1 shows the parameters of all convolutional layers.

## 4. Results

To verify the effectiveness of our proposed TID, we chose the indoor training SET (ITS), synthetic objective testing set (SOTS), and hybrid subjective testing set (HSTS) in RESIDE [42] for the experiments. Among them, ITS was used as the training set, and SOTS and HSTS were used as the test set.

### 4.1. Comparison with State-of-the-Art Methods

We comprehensively performed comparisons with several state-of-the-art image dehazing methods. The compared methods are Fattal’s [26], FVR [43], DehazeNet [32], AOD-Net [15], EPDN [35], and AECR-Net [36]. We used the metrics PSNR and SSIM [44] to evaluate the quality of restored images.

#### 4.1.1. Quantitative and Qualitative Results on the Synthetic Dataset

In our experiment, we selected 500 and 10 images from SOTS and HSTS, respectively. All selected images are synthetic hazy images with haze-free ground truth.

The second row in Table 2 shows the quantitative evaluation of SOTS. Compared with these state-of-the-art methods, our method has a significant improvement in both PSNR and SSIM evaluation indicators. Furthermore, we also tested on HSTS outdoor synthetic data. As shown in the third row in Table 2, our proposed method also achieves better PSNR and SSIM results than other methods on the HSTS dataset.

**Figure 4:**
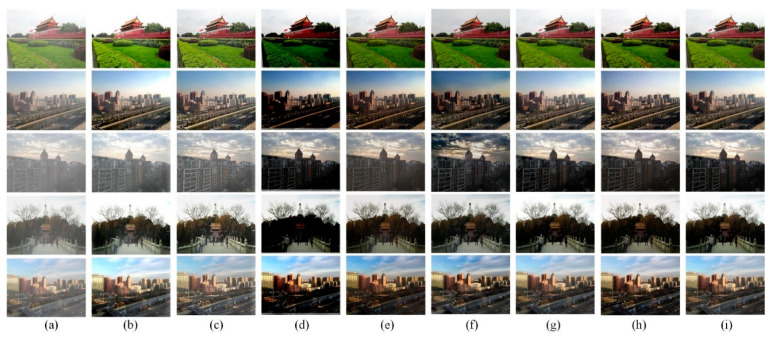
Dehazing results on HSTS dataset. (**a**) Hazy image; (**b**) Fattal’s [26]; (**c**) FVR [43]; (**d**) DehazeNet [32]; (**e**) AOD-Net [15]; (**f**) EPDN [35]; (**g**) AECR-Net [36]; (**h**) TID (ours); (**i**) ground truth.

Figure 4 shows some dehazing images from the HSTS dataset. As shown in Figure 4b,c, the prior-based methods do not perform well. For the learning-based methods, as shown in Figure 4d, the result generated by DehazeNet [32] is significantly darker. As shown in Figure 4e, although the recovered images obtained by AOD-Net [15] have higher brightness, the ability to restore details is poor. For example, the city wall and the red flags on the city wall in the first image, the bridge deck position in the fourth image, and the overall dehazing effect of the third image are not good. As shown in Figure 4f, the result obtained by EPDN [35] is close to the ground truth image, but its effect is not good in the sky area. As shown in Figure 4g, the result obtained by AECR-Net [36] is also close to the ground truth images, but its generalization ability to real-world images is not good, which we will discuss in Section 4.1.2. The recovered image obtained by our proposed method is visually closer to the haze-free ground truth than other methods.

#### 4.1.2. Qualitative Results in Real-World Hazy Images

**Figure 5:**
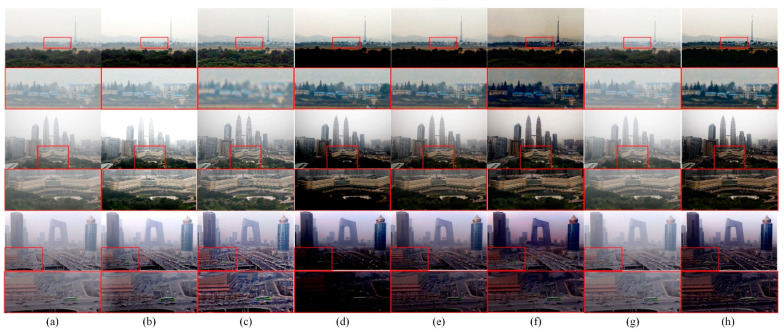
Dehazing results on HSTS dataset. (**a**) Hazy image; (**b**) Fattal’s [26]; (**c**) FVR [43]; (**d**) DehazeNet [32]; (**e**) AOD-Net [15]; (**f**) EPDN [26]; (**g**) AECR-Net [36]; (**h**) TID (ours).

To evaluate the generalization ability of our proposed method, we selected 10 real-world hazy images (without haze-free ground truth) from HSTS. As shown in Figure 5b, Fattal’s [26] method is less effective than other methods. As shown in Figure 5b,c, the prior-based method does not perform well in details, especially in the red frame area of the first image. As shown in Figure 5d, DehazeNet [32] has too-low brightness in the red frame area of the third image. As shown in Figure 5e, AOD-Net [15] performs well in real-world images, but it is inferior to our proposed method in detail. As shown in Figure 5f, EPDN [35] performs better in the red frame area but has severe color distortion in the sky area, such as in the first and third images. As shown in Figure 5g, AECR-Net [36] performs poorly overall in real-world images and has inferior generalization ability. In summary, our method can generalize well on real-world hazy images in visual quality and better preserves detailed information, especially in the area shown in the red box.

### 4.2. Ablation Studies

In order to verify the effectiveness of our proposed attention module, we designed two ablation studies: (1) attention module and non-attention module; (2) our proposed attention module compared with SE-Net [19] and CBAM [18]; (3) for the output of TCAM, the average-pooling patch token compared with the class token.

#### 4.2.1. Attention Module and Non-Attention Module

**Figure 6:**
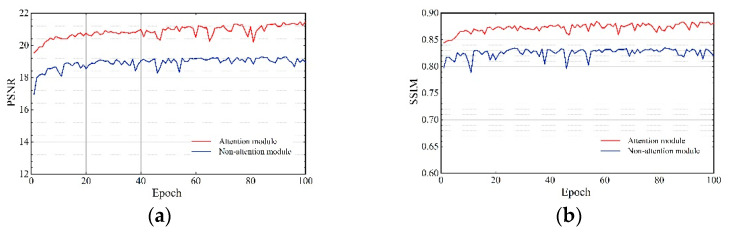
Effects of ablation study (1).

We removed the attention module in our proposed network architecture. Then the two networks were quantitatively analyzed using PSNR and SSIM on the SOTS test set. Figure 6 shows the validation curve, compared with the non-attention module. The validation curve with the attention module has a smaller oscillation amplitude and a better fit effect. Table 3 shows the quantitative results of SOTS. The PSNR of using the attention module is 6.89% higher than that of the non-attention module, and the SSIM is 3.61% higher. This ablation study demonstrates that the network with attention modules can effectively improve performance haze removal.

#### 4.2.2. Our Proposed Attention Module Is Compared with SE-Net and CBAM

**Figure 7:**
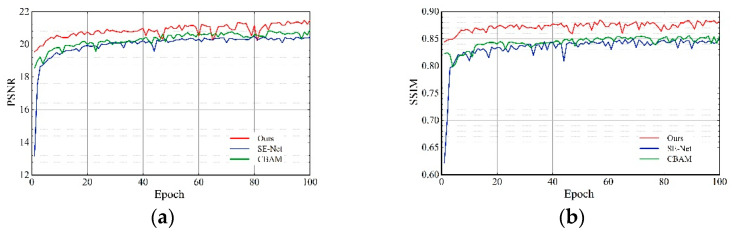
Effects of ablation study (2).

We used SE-Net [19] and CBAM [18] instead of the attention module we proposed and also performed quantitative analysis on these three networks. As shown in Figure 7 and Table 4, compared with SE-Net [19], the PSNR of our proposed attention module is 6.05% higher, and SSIM is 2.80% higher; compared to CBAM [18], PSNR is 3.29% higher, and SSIM is 3.00% higher. This demonstrates that the attention module we proposed is better than the SE-Net [19] and CBAM [18] performance.

#### 4.2.3. For Output of TCAM, the Average-Pooled Patch Token Is Compared with the Class Token

**Figure 8:**
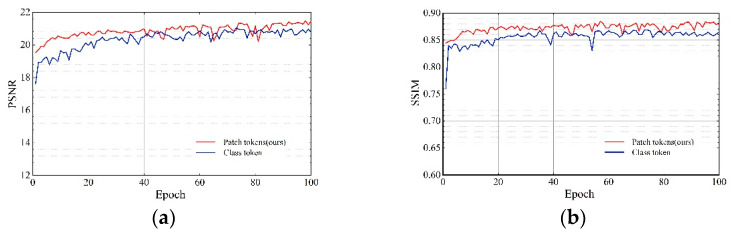
Effects of ablation study (3).

For the output of TCAM, we used the class token instead of the average-pooled patch token. Then, a quantitative analysis of these two networks was performed. As shown in Figure 8 and Table 5, compared with the class token, the PSNR of the average-pooled patch token is 1.92% higher, and SSIM is 1.88% higher. This ablation study demonstrates that the average-pooled patch token is more effective than the class token.

## 5. Conclusions

In this paper, we propose a Transformer-based channel attention module (TCAM) combined with a spatial attention module to enhance a CNN-based backbone network. Our proposed TCAM utilizes Transformer to address the limitation of the local receptive fields of CNNs, and then uses a spatial attention module as its complement, which finally achieves enhanced detailed information of feature maps along both the channel and spatial dimensions. At the same time, we use a multiscale parallel residual module to extract features of different scales to achieve feature reuse. We perform quantitative and qualitative evaluations against state-of-the-art methods on SOTS and HSTS datasets. Experimental results show that our proposed method has superior performance. Compared with AECR-Net, our proposed method improves PSNR by 4.34% and 4.64% and SSIM by 2.41% and 1.21% in SOTS and HSTS, respectively.

In addition, we designed three ablation studies to verify our proposed attention module. The results of comprehensive ablation experiments show that our proposed attention module can improve image dehazing performance and outperform existing attention modules.

## Figures and Tables

**Table 1:** Parameter of the convolution layer.

Layer	Kernel_Size/Padding	Output Channel
Conv1_1	1 × 1/0	3
Conv1_2	3 × 3/1	3
Conv1_3	5 × 5/2	9
Conv2_1	3 × 3/1	3
Conv2_2	5 × 5/2	3
Conv2_3	7 × 7/3	9
Conv3	3 × 3/1	3

**Table 2:** Average PSNR/SSIM of dehazed results on the SOTS and HSTS dataset.

Dataset		Fattal’s	FVR	DehazeNet	AOD-Net	EPDN	AECR-Net	Ours
SOTS	PSNR	161143	16.8931	18.7453	18.5211	20.1722	20.5466	21.4393
	SSIM	0.7261	0.7484	0.8314	0.8314	0.8576	0.8642	0.8851
HSTS	PSNR	17.7348	18.0142	21.2218	21.2218	22.3145	22.7693	23.8276
	SSIM	0.8123	0.8217	0.8687	0.8687	0.8809	0.8914	0.9022

**Table 3:** Average PSNR/SSIM of ablation study (1).

	Non-Attention Module	Attention Module
PSNR	20.0566	21.4394
SSIM	0.8543	0.8851

**Table 4:** Average PSNR/SSIM of ablation study (2).

	SE-Net	CBAM	Ours
PSNR	20.2163	20.7560	21.4394
SSIM	0.8610	0.8593	0.8851

**Table 5:** Average PSNR/SSIM of ablation study (3).

	Non-Attention Module	Attention Module
PSNR	21.0335	21.4394
SSIM	0.8688	0.8851

## Data Availability

Data underlying the results presented in this paper are not publicly available at this time but may be obtained from the authors upon reasonable request.
